# Primary Analysis of the Expressed Sequence Tags in a Pentastomid Nymph cDNA Library

**DOI:** 10.1371/journal.pone.0056511

**Published:** 2013-02-20

**Authors:** Jing Zhang, Yujuan Shen, Zhongying Yuan, Jianhai Yin, Wei Zang, Yuxin Xu, Weiyuan Lu, Yanjuan Wang, Ying Wang, Jianping Cao

**Affiliations:** National Institute of Parasitic Diseases, Chinese Center for Disease Control and Prevention, Key Laboratory of Parasite and Vector Biology, Ministry of Health, World Health Organization Collaborating Center for Malaria, Schistosomiasis and Filariasis, Shanghai, People’s Republic of China; Queensland Institute of Medical Research, Australia

## Abstract

**Background:**

Pentastomiasis is a rare zoonotic disease caused by pentastomids. Despite their worm-like appearance, they are commonly placed into a separate sub-class of the subphylum Crustacea, phylum Arthropoda. However, until now, the systematic classification of the pentastomids and the diagnosis of pentastomiasis are immature, and genetic information about *pentastomid* nylum is almost nonexistent. The objective of this study was to obtain information on pentastomid nymph genes and identify the gene homologues related to host-parasite interactions or stage-specific antigens.

**Methodology/Principal Findings:**

Total pentastomid nymph RNA was used to construct a cDNA library and 500 colonies were sequenced. Analysis shows one hundred and ninety-seven unigenes were identified. In which, 147 genes were annotated, and 75 unigenes (53.19%) were mapped to 82 KEGG pathways, including 29 metabolism pathways, 29 genetic information processing pathways, 4 environmental information processing pathways, 7 cell motility pathways and 5 organismal systems pathways. Additionally, two host-parasite interaction-related gene homologues**,** a putative Kunitz inhibitor and a putative cysteine protease.

**Conclusion/Significance:**

We first successfully constructed a cDNA library and gained a number of expressed sequence tags (EST) from pentastomid nymphs, which will lay the foundation for the further study on pentastomids and pentastomiasis.

## Introduction

Pentastomids, a group of vermiform animals related to both arthropods and crustaceans, cause the zoonotic disease pentastomiasis [1]. Pentastomids are commonly classed into a separate sub-class of the subphylum Crustacea, phylum Arthropoda. But them have worm-like appearance [2]. Pentastomids are invertebrate endoparasites which have a wide range of hosts including reptiles, birds and mammals [2]–[4]. The adults mainly inhabit the respiratory systems of their hosts, and the larvae and nymphs can settle in any organ of the host. There are two main genera of pentastomids involved in human infection: *Linguatula serrata* and *Armillifer armillatus* [5], [6].

The most common forms of human pentastomiasis can be classified into two major types: visceral pentastomiasis is caused by ingesting infective eggs of pentastomids, when humans act as an intermediate host; whereas nasopharyngeal pentastomiasis occurs when uncooked tissue containing encysted nymphs of *L. serrata* is ingested, with humans serving as an aberrant definitive host. Infection with pentastomids is mostly asymptomatic in humans [7], [8] and large numbers of larvae can inhibit a human host without causing obstruction, damage or a significant immune response. However, pentastomids have the potential to cause bodily harm, and in extreme cases, medical emergencies [1].

As pentastomids do not trigger a massive specific immune reaction, serologic tests are difficult to perform [1]. Gene trait analysis of pathogens provides a feasible approach to classify and diagnose disease; however, genetic information on pentastomids is almost nonexistent. Although scientists have made significant breakthroughs in this field in the past few decades, the classification and diagnosis of pentastomiasis remains difficult. As pentastomiasis is mostly asymptomatic, the disease often goes undetected until an autopsy is performed after the death of the host [7], [9], [10].

In the present study, we aimed to gain genetic information by constructing a pentastomid nymph cDNA library. This work has significance for the classification and diagnosis of pentastomiasis, and will lay the foundation for future functional genomic studies of pentastomid nymphs.

## Materials and Methods

### Parasite Culture

Kunming mice were infected with the eggs of *Armillifer agkistrodontis* from Agkistrodon acutus snakes and euthanized one and a half years later. Pentastomids at the larval stage were isolated. All animal procedures performed in this study were conducted in accordance with, and by approval of, the Laboratory Animal Welfare & Ethics Committee (LAWEC), National Institute of Parasitic Diseases, Chinese Center for Diseases Control and Prevention (Permit Number: IPD 2009-8).

### Preparation of High Quality mRNA

High quality total mRNA was isolated from 3.5 g of whole fresh pentastomid nymph tissue using the FastTrack® 2.0 mRNA Isolation Kit (Invitrogen, Carlsbad, CA, USA) according to the manufacturer’s instructions. The quality and concentration of the RNA samples were confirmed by gel electrophoresis using denaturting formaldehyde agarose gels.

### cDNA Library Construction and Sequencing

The cDNA library was constructed using the SuperScript™ Plasmid System with Gateway® Technology for cDNA Synthesis and Cloning Kit (Invitrogen, Carlsbad, CA, USA). Briefly, 2 µg mRNA was used as a template for the synthesis of first strand cDNA using a *Not* I primer-adapter and second strand cDNA was synthesized using T4 DNA polymerase. A *Sal* I adapter was added to the ends of the cDNA fragments using T4 DNA ligase, the cDNA was digested using *Not* I, the different sticky ends of the cDNA fragments were cloned into the plasmid pEXP-AD502 which had been digested with *Sal* I and *Not* I, and then transformed into *E. coli*. Aliquots of the transformed cells were plated onto selective agar containing 100 µg/ml ampicillin, incubated overnight at 37°C and single colonies were picked. Wells in a 96-well microplate were filled with randomly selected single plaques containing recombinant clones. Subsequently, using the T3 primer and ABI™ PRISM® BigDye Terminator Cycle Sequencing Ready Reaction Kit(Applied Biosystems, Foster City, CA, USA), single-pass nucleotide sequencing of the 5′-end of each cDNA fragment was performed. The sequencing products were analyzed by ABI™ PRISM® 377 DNA Sequencer (Applied Biosystems, Foster City, CA, USA).

### Sequence Analysis

First, the low quality sequences and the contamination from vectors were omitted. Meanwhile, all sequences shorter than 100 bp were re-moved by way of scripts programmed in Perl language. And then the high-quality ESTs were assembled into contigs through Phrap software. Second, all unique-contigs were anonated through Blastx and Blastn(NCBI), respectively(E-value<10^−5^). Gene ontology (GO) analysis [11], [12] was used to analyze the main function of the genes, according to the the key functional GO classifications in the NCBI database. Generally, Fisher’s Exact Test was used to classify the GO category, and the False Discovery Rate (FDR) was calculated to correct the *P*-value (A smaller FDR represents a smaller error in determination of the *P*-value). Similarly, pathway analysis was used to determine which pathways the genes participate in, according to the Kyoto Encyclopedia of Genes and Genomes (KEGG) [13], [14]. The Fisher’s Exact Test was used to select significant pathways, the threshold of significance was defined by the *P*-value and FDR, and then the degree of enrichment was calculated.

## Results

### Construction of the Pentastomid Nymph cDNA Library

Total pentastomid nymph RNA was used to construct a cDNA library. The titres of the primary and amplified libraries were 2.0×10^6^ pfu/ml and 2.5×10^9^ pfu/ml, respectively. The recombination efficiency of the amplified library was 85%. The size of the inserted cDNA fragments ranged from 200 to 1800 bp. Together, these criteria indicated the construction of a high quality cDNA library.

### Sequencing and Contig Assembly

**Figure 1:**
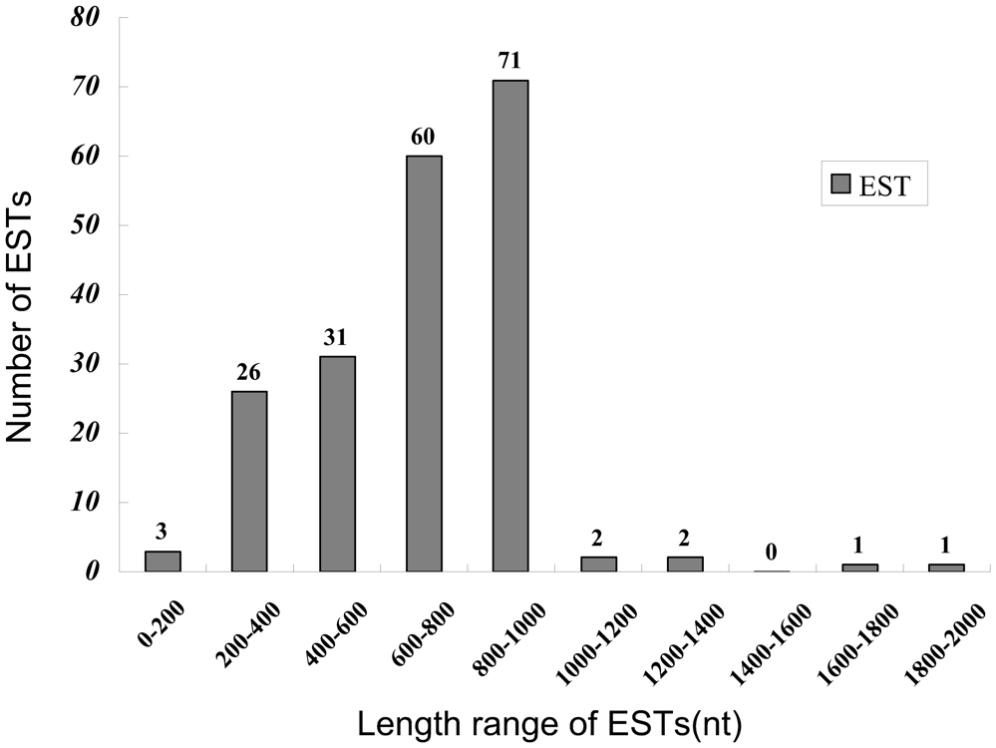
Distribution of the sequence length of the 197 unigenes in the pentastomid nymph cDNA library.

In this study, a total of 512 clones were sequenced, of which 388 sequences were valid, and 197 unigenes (27 contigs and 170 singlets) were identified through alignment and sequence assembly. The length of the unigenes ranged from 76 bp to 1926 bp, with an average length of 600 bp (**Table 1** **;** **Fig. 1**).

**Table 1:** Summary of the pentastomid nymph cDNA library analysis.

Description	Number
Total number of sequences	512
Number of valid sequences	388
Average expressed sequence tag (EST) length (nt)	600
Number of unigenes	197
Number of contigs	27
Number of singlets	170
Number of annotated genes	141

### In-depth Analysis

**Figure 2:**
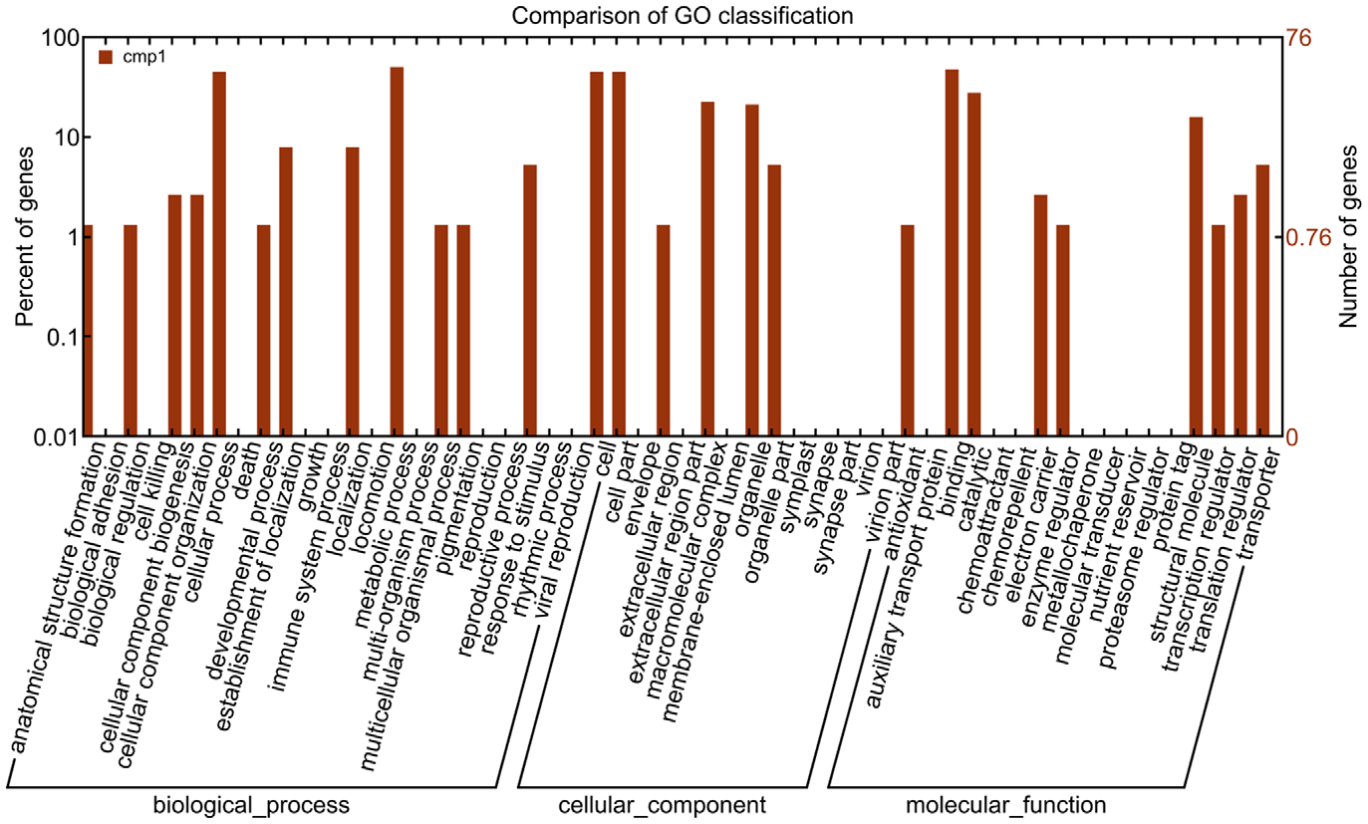
Functional annotation of the pentastomid nymph cDNA library unigenes, based on the Uniprot database. Gene Ontology (GO) terms at the 2nd level are plotted. “Biological process”, “Cellular component” and “Molecular function” are categorized independently in this ontology.

In order to obtain more information on the 197 unigenes, we performed an in-depth analysis of the sequences using BLASTX (InterPro, KEGG, Swissprot and TrEMBL). As shown in **Table 1**, 141 unigenes (∼71%) were successfully annotated; including 27 highly expressed genes (**Table 2**). The 141 sequences were annotated and classified according to their functions by GO analysis. As shown in **Fig. 2**, the 141 annotated unigenes are involved in 54 functions, and could be classified into three major groups: biological-processes (6.09%), cellular-components (5.58%) and molecular-functions (26.90%). Within the category “Molecular Function”, the subcategories “auxiliary transport protein”, “binding” and “protein tag” contained the highest number of unigenes, and the majority of gene functions were related to the regulation of protein activity.

**Table 2:** The most abundant transcripts in the pentastomid nymph cDNA library.

Transcript	Length (bp)	Description
Contig1	839	B2GUX7_RAT Cellular repressor of E1A
Contig2	675	Cytochrome c oxidase subunit II C
Contig3	657	No significant similarity found
Contig4	952	No significant similarity found
Contig5	1926	Hypothetical protein
Contig6	442	Cytochrome b/b6
Contig7	1288	Hypothetical protein
Contig8	629	No significant similarity found
Contig9	1346	Hypothetical protein
Contig10	705	No significant similarity found
Contig11	454	Hypothetical protein
Contig12	760	NADH:ubiquinone/plastoquinone oxidoreductase, chain 3
Contig13	623	Heat shock protein Hsp20
Contig14	1652	Gold
Contig15	828	Smooth muscle protein/calponin
Contig16	1147	Enhancer of rudimentary
Contig17	813	No significant similarity found
Contig18	699	Heat shock protein Hsp20
Contig19	1164	Cysteine type peptidase
Contig20	917	FAS1 domain
Contig21	924	No significant similarity found
Contig22	295	No significant similarity found
Contig23	984	Hypothetical protein
Contig24	944	Hypothetical protein
Contig25	600	No significant similarity found
Contig26	721	Proteinase inhibitor I2, Kunitz metazoa
Contig27	933	No significant similarity found

To further explore the function of the annotated genes, pathway analysis was performed using KEGG. A total of 75 unigenes (53.19%) were mapped to 82 KEGG pathways, including 29 metabolism pathways, 29 genetic information processing pathways, four environmental information processing pathways, seven cell motility pathways and five organismal systems pathways. **Table 3** lists the top 20 annotated metabolic pathways. It was noted that ribosomal, oxidative phosphorylation, endoplasmic reticulum protein processing and the insulin signaling pathways were most active in pentastomids at the nymph stage.

**Table 3:** Top 20 metabolic pathways in the pentastomid nymph cDNA library, mapped using Kyoto Encyclopedia of Genes and Genomes (KEGG) pathway analysis.

KEGG	No. of clusters	Pathway
map03010	13	Ribosome
map00190	6	Oxidative phosphorylation
map04141	4	Protein processing in endoplasmic reticulum
map04910	4	Insulin signaling pathway
map00230	3	Purine metabolism
map00240	3	Pyrimidine metabolism
map04142	3	Lysosome
map04144	3	Endocytosis
map04150	3	mTOR signaling pathway
map04260	3	Cardiac muscle contraction
map04612	3	Antigen processing and presentation
map04810	3	Regulation of actin cytoskeleton
map00010	2	Glycolysis/Gluconeogenesis
map00480	2	Glutathione metabolism
map03020	2	RNA polymerase
map03320	2	PPAR signaling pathway
map04270	2	Vascular smooth muscle contraction
map04666	2	Fc gamma R-mediated phagocytosis
map04720	2	Long-term potentiation
map05130	2	Pathogenic Escherichia coli infection

### Identification of Host-parasite Interaction-related Gene Homologues

**Figure 3:**
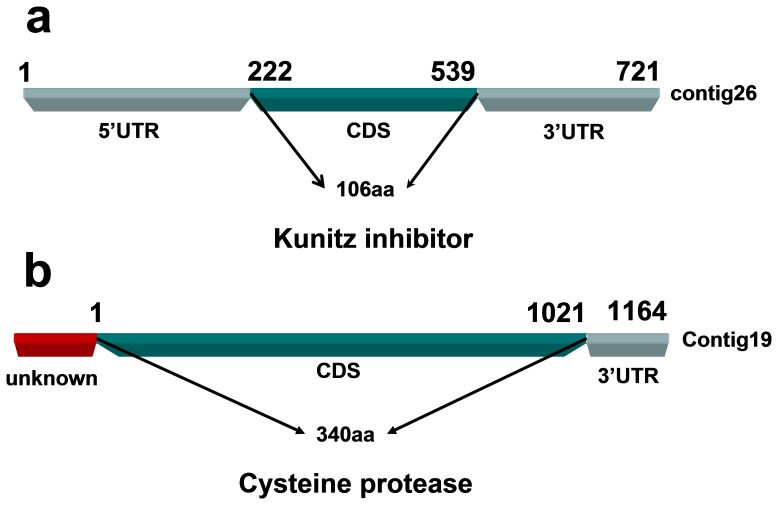
Gene architecture of the pentastomid nymph host-parasite interaction-related gene homologues. Schematic diagram of (a) the putative Kunitz inhibitor and (b) the putative cysteine protease identified from the cDNA library.

Of the 197 unigenes, Contig26 was expressed at the highest levels. As shown in Fig. 3a, the complete mRNA of the Contig26 gene is 721 bp in length. The sequenced coding regions (CDS) of this gene ranged from 222 bp to 539 bp, and the gene encodes a protein of 106 amino acids. Multiple sequence alignments revealed that the gene shared a highly homologous domain with the Kunitz inhibitors, which are known to participate in the infection process of parasites ([15], [16]).

In addition, another interesting gene, contig19, was identified. We obtained a partial mRNA sequence for this gene. As shown in **Fig. 3b**, the known sequence is 1164 bp in size, including the partial CDS and complete 3` untranslated region (UTR). The known fragment encodes a protein of 340 amino acids, which shares a high degree of homology with the cysteine protease family. The cysteine proteases of trematode parasites play an essential role in parasite physiology ([17], [18]).

## Discussion

Pentastomidosis is a zoonotic parasitic disease. In the past, pentastomidosis was considered to be a rare disease; however, the number of reported cases has increased significantly in recent years, which has lead to heightened concern within the medical profession. The optimal diagnostic techniques and therapeutic approach for pentastomidosis are still lacking, despite the fact this disease was initially described nearly 165 years ago [19]. Therefore, further research on pentastomids, the pathogens responsible for pentastomidosis, is urgently required to improve the prevention, diagnosis and treatment of this disease.

In this study, we constructed a pentastomid nymph cDNA library. The titre (2.5×10^9 ^pfu/ml), recombination efficiency (85%) and inserted fragment length (200 to 1800 bp) demonstrated that a high quality cDNA library was successfully constructed [20], [21]. Furthermore, 197 unigenes (170 singlets and 27 contigs) were obtained through sequencing and bioinformatic analysis. Most of the identified genes are involved in the regulation of protein activity. Twenty seven genes (Contig1–Contig27) were expressed at high levels, each with a copy number greater than one. Therefore, we speculate that these highly expressed unigenes play vital roles in pentastomids at the nymph stage. Pathway analysis revealed that the 75 mapped unigenes are involved in 82 KEGG pathways, including 29 metabolism pathways, 29 genetic information processing pathways, four environmental information processing pathways, seven cell motility pathways and five organismal systems pathways.

From the ESTs, we identified two host-parasite interaction-related gene homologues (Contig26 and Contig19). Contig26 encodes a protein homologous to a Kunitz inhibitor. Kunitz inhibitors are a class of serine protease inhibitors [22], [23], which are reported to play a very important role at the onset of infection [15], [16]. Thus, we propose that this gene may have the potential to act at the pentastomid interface, and interfere with host physiological processes at the infection stage. Contig19 encodes a cysteine protease, and cysteine proteases are known to play an essential role in life of trematode parasites, involving the turnover of parasite proteins, parasite penetration and invasion, hydrolysis of host proteins for nutrient uptake and modulation of the host immune system [17], [18]. High levels of the putative cysteine protease were observed during the infection stage in the pentastomid nymph cDNA library, indicating that Contig19 may be associated with nutritional requirements in pentastomids at the infection stage.

In conclusion, we successfully constructed a cDNA library and identified 197 pentastomid nymph unigenes. Furthermore, we performed primary analysis of these genes using a bioinformatics approach. This study has laid the foundation for further study of pentastomids.
